# Catechin Augments the Antifungal Efficacy of Fluconazole Against *Candida parapsilosis*

**DOI:** 10.3390/ijms27020620

**Published:** 2026-01-07

**Authors:** Nora Tóth Hervay, Alexandra Konečná, Daniel Eliaš, Petra Kocúreková, Juraj Jacko, Hanka Súlovská, Libuša Šikurová, Yvetta Gbelská

**Affiliations:** 1 Department of Microbiology and Virology, Faculty of Natural Sciences, Comenius University in Bratislava, Ilkovicova 6, 842 15 Bratislava, Slovakia; 2 Department of Nuclear Physics and Biophysics, Faculty of Mathematics, Physics and Informatics, Comenius University in Bratislava, 842 48 Bratislava, Slovakia

**Keywords:** *Candida parapsilosis*, fluconazole, catechin, ROS

## Abstract

The rising global incidence of *Candida parapsilosis* infections is increasingly complicated by antifungal resistance, resulting in frequent therapeutic failure. This study investigated the potential of the natural compound catechin to enhance the efficacy of fluconazole through synergistic interaction. We evaluated the susceptibility of *C. parapsilosis* clinical isolates and a reference strain to combinations of catechin and fluconazole using standardized microbiological assays and molecular techniques. In vivo efficacy was assessed using the *Galleria mellonella* infection model. Mechanistic studies included the measurement of intracellular reactive oxygen species (ROS) production and plasma membrane permeability. Catechin alone caused growth retardation in all strains. However, the combination of catechin and fluconazole resulted in complete growth inhibition of the reference strain and significant growth reduction in azole-resistant clinical isolates. While the combination slightly increased intracellular ROS production, no significant changes in plasma membrane permeability or membrane potential were observed. Notably, catechin induced the expression of the resistance-associated genes *CpTAC1* and *CpCDR1B* in resistant isolates. In vivo experiments demonstrated that catechin significantly reduced mortality in *G. mellonella* larvae infected with *C. parapsilosis*. These findings suggest that catechin is a promising candidate for developing synergistic antifungal therapies against resistant *Candida* species.

## 1. Introduction

Most fungi belonging to *Candida* species are clinically relevant pathogens. The majority of them are opportunistic, existing in the environment or as commensals, but able to cause invasive infections in immunocompromised individuals [1]. Although *Candida albicans* is the most prevalent species associated with infections, a dramatic rise in drug-resistant infections caused by non-albicans species has recently occurred. Among these, *Candida parapsilosis* infections have become particularly concerning due to a sharp increase in azole resistance and numerous fluconazole-resistant nosocomial outbreaks [2,3]. The *C. parapsilosis* complex comprises the non-pathogenic species *Candida margitis* and three other opportunistic, but clinically less important, species able to cause infections, *Candida orthopsilosis*, *Candida metapsilosis* and *Candida theae*. Of these species, *C. parapsilosis* is the most prevalent infectious agent that has gone from being historically associated with pediatric infections to a frequent cause of catheter-associated bloodstream infections in adults [4,5].

The clinical persistence and environmental adaptability of this pathogen are supported by its specific genetic architecture and virulence factors. *C. parapsilosis* is a diploid yeast species with a highly homozygous genome (13.13 Mb) organized into eight chromosome pairs containing approximately 5.837 protein-coding genes. *C. parapsilosis* possesses 30.9 kbp long linear mitochondrial DNA terminating on both sides with specific telomeric structures, proposed to be a potential molecular marker for clinical diagnostics [6]. *C. parapsilosis* belongs to the CTG clade species that translates CTG as serine rather than leucine. Recent global analyses of sequenced clinical and environmental isolates revealed very low sequence divergence but a high variation in the copy number of key genes encoding agglutinin-like sequence (Als) proteins, enabling adhesion to host epithelial cells [7]. Although *C. parapsilosis* forms less complex and thinner biofilms than *C. albicans*, these biofilms are readily formed in the presence of high glucose or lipid-rich media. Their presence on plastic and medically implanted devices remains a major source of infection.

Recent surveillance has highlighted increasing rates of fluconazole resistance in *C. parapsilosis* isolates worldwide [8,9,10]. Mutations in the zinc-finger transcriptional activator *Cp*Tac1p have been associated with fluconazole resistance in *C. parapsilosis* clinical isolates, leading to the overexpression of ABC transporter-encoding genes such as *CpCDR1*, *CpCDR1B* or *CpCDR1C* [11]. Gain-of-function mutations in the *MRR1* gene, which encodes another key transcription factor, can result in transcriptional activation of the *MDR1* gene, leading to high levels of drug efflux and clinical fluconazole resistance [12]. The ATP-binding cassette (ABC) and major facilitator superfamily (MFS) transporters are known to be responsible for lowering the accumulation of azoles inside the *C. albicans* yeast cell by actively translocating compounds across the cell membrane [13]. In *C. parapsilosis*, the “MFS signaling pathway” is primarily defined by the Mrr1-MDR regulatory axis. While earlier models often separated the ABC (Tac1-CDR) and MFS (Mrr1-MDR) pathways, recent research indicates a significant level of coordinated regulation with both pathways operating simultaneously to achieve clinical resistance [11,12]. Research by Franconi et al. [14] highlights that azole resistance in fungi is driven by diverse genetic mechanisms beyond simple point mutations. These mechanisms involve large-scale genomic changes that alter the expression or function of key resistance genes, such as those encoding the target enzyme *ERG11* and various drug efflux pumps. Due to its environmental persistence, increasing incidence of fluconazole resistance, reduced susceptibility to echinocandins and high mortality rates, *C. parapsilosis* has been included in the World Health Organization’s priority pathogen list [15]. The clinically available armamentarium for antifungal treatment is very scarce. Therefore, there is an urgent need for novel antifungal strategies. One promising approach to expand the antifungal spectrum, decrease inhibitory dosages, reduce toxicity and prevent the development of resistance is to expand the potential of existing antifungals through drug synergy. Several reports point to the increased efficacy of antifungal azoles when combined with catechins [16,17,18]. Catechin represents the family name of compounds derived from catechin, which are distributed in a variety of foods and herbs, including tea, apples, persimmons, cacaos, grapes and berries. A catechin is a polyphenol compound with diverse biological activities, including antimicrobial and antioxidant properties [19,20].

In our previous studies, we have shown that catechin potentiates the activity of miconazole in both a *Nakaseomyces glabratus* (previously known as *Candida glabrata*) laboratory strain and *N. glabratus* clinical isolates [21,22]. The main aim of this study is to evaluate the synergistic potential of combining the natural compound catechin with the conventional antifungal drug fluconazole to improve the treatment of resistant *C. parapsilosis* infections.

## 2. Results

In this work, we investigated the effect of catechin on modulating azole susceptibility in two *C. parapsilosis* clinical isolates originating from the hemoculture (HC) and central venous catheter (CVC) of the same patient [23].

### 2.1. Catechin Enhances the Antifungal Activity of Fluconazole in C. parapsilosis

**Figure 1:**
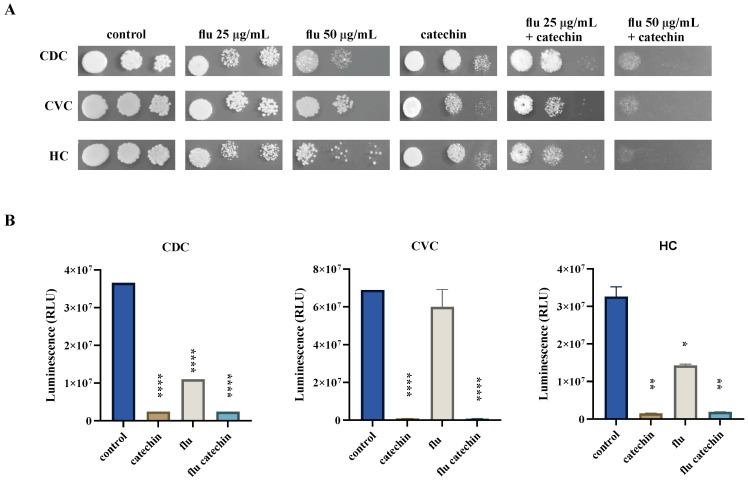
Catechin affects the growth and viability of *C. parapsilosis* strains. Growth of *C. parapsilosis* reference strain CDC317 (CDC) and the clinical isolates from CVC and HC on the YPD medium with fluconazole (flu) alone and in combination with catechin (2 mg/mL) (**A**). Cells were spotted as tenfold dilution series (10^7^, 10^6^ and 10^5^ cells/mL) on YPD plates and incubated at 35 °C for 2 days. BacTiter-Glo™ Microbial Cell Viability Assay (**B**). Luminescence recorded after incubation of cells in YPD broth with fluconazole, (8 μg/mL), catechin (2 mg/mL) or with their combination. * *p* < 0.050 statistically significant; ** *p* < 0.010 highly significant; **** *p* < 0.0001 highly extremely significant. RLU relative luminescence unit.

To explore the ability of catechin to enhance the antifungal effect of fluconazole, we performed spot assays and a cell viability assay based on the quantification of the ATP present. Figure 1A shows that the combination of fluconazole with catechin leads to growth inhibition in both *C. parapsilosis* clinical isolates as well as in the *C. parapsilosis* CDC317 that was used as a reference strain. The results of the spot assays were corroborated by the cell viability assay shown in Figure 1B. Although the cell viability decreased in the presence of fluconazole in all three strains tested, the simultaneous presence of catechin and fluconazole led to a complete loss of cell viability.

### 2.2. Catechin Induces Intracellular Reactive Oxygen Species (ROS) Production in C. parapsilosis

**Figure 2:**
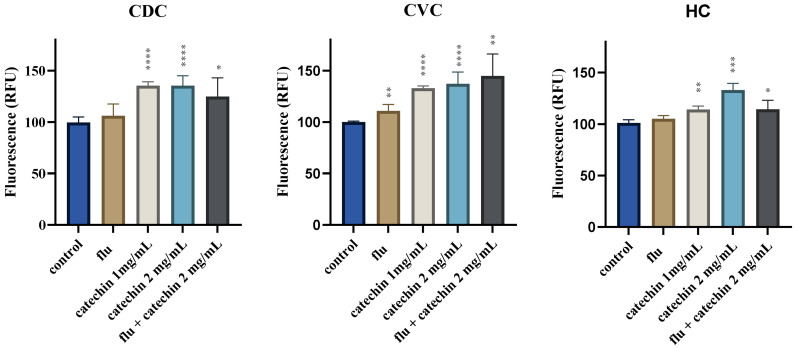
Production of ROS by *C. parapsilosis* reference strain CDC317 (CDC) and two clinical isolates (CVC and HC) in the presence of fluconazole (5 μg/mL) (flu), catechin (1 mg/mL, 2 mg/mL) or their combination. Values represent the mean of 3 biological replicas. *p* < 0.050 (*), *p* < 0.010 (**), *p* < 0.001 (***) and *p* < 0.0001 (****) were considered statistically significant. RFU relative fluorescence unit.

To see whether the susceptibility profile of the *C. parapsilosis* clinical isolates could be influenced by the pro-oxidant activity of catechin, we examined intracellular ROS production in *C. parapsilosis* cells. The presence of intracellular ROS during catechin exposure was assessed using the fluorescent probe H2DCFDA (2′,7′-dichlorodihydrofluorescein diacetate). In the presence of catechin alone, a slight increase in the intracellular ROS concentration was observed in the cells of all three *C. parapsilosis* strains tested (Figure 2).

### 2.3. Expression of Genes Involved in C. parapsilosis Azole Resistance

Although we did not observe any statistically significant effect of catechin or its combination with fluconazole on plasma membrane fluidity or plasma membrane potential in the *C. parapsilosis* strains studied (Figure S1, Table S3), in the next experiment we looked for genes differentially expressed in the presence of catechin. We subjected the *C. parapsilosis* clinical isolates and the reference strain to transcriptional profiling by RT-PCR to assess changes in the expression of genes associated with fluconazole resistance in *C. parapsilosis.*

**Figure 3:**
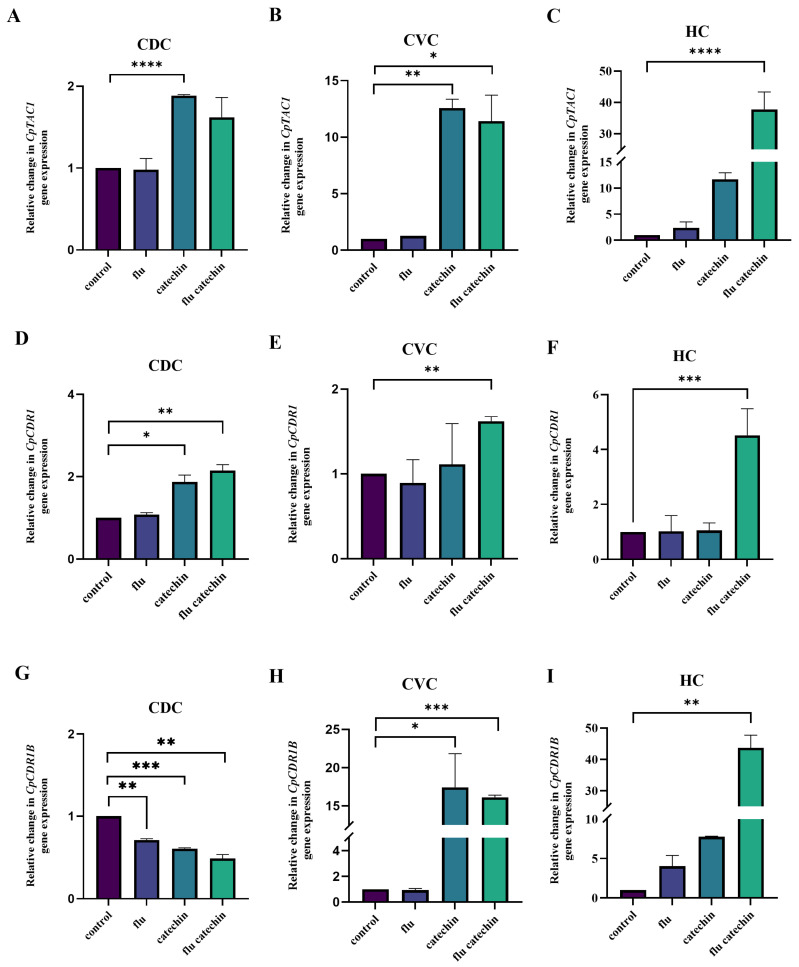
Relative gene expression levels of genes encoding transcription factor *Cp*Tac1p (**A**–**C**) and transport proteins (**D**–**I**) in the *C. parapsilosis* strains. The results are expressed as mean values of three independent experiments (±standard deviation) normalized to the *CpACT1* gene expression level. Figure shows the relative changes in gene expression after 2 h of incubation in the presence of fluconazole 5 μg/mL (flu), catechin 2 mg/mL or their combination compared to untreated samples set as 1. * *p* < 0.050 statistically significant; ** *p* < 0.010 highly significant; *** *p* < 0.001 extremely significant; **** *p* < 0.0001 highly extremely significant.

**Figure 4:**
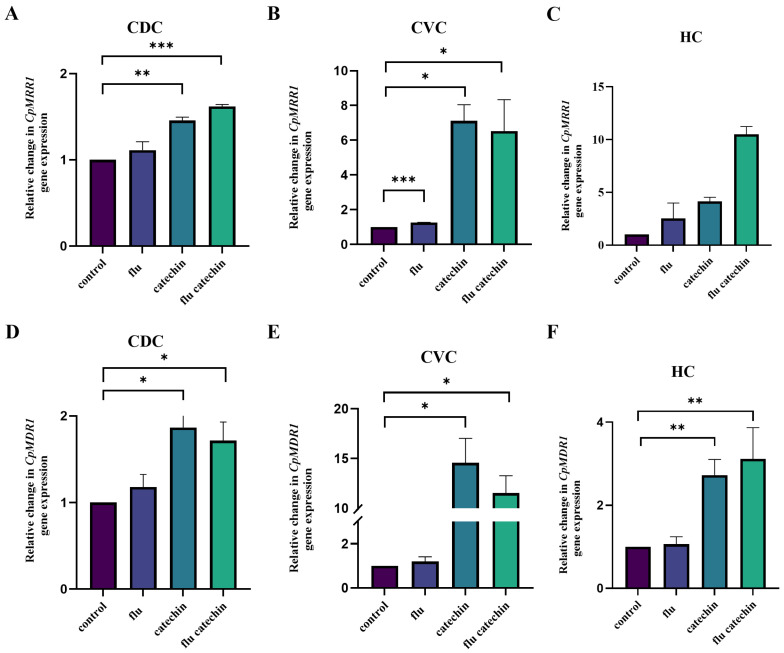
Relative gene expression levels of genes encoding transcription factor *Cp*Mrr1p (**A**–**C**) and transport protein *Cp*Mdr1p (**D**–**F**) in the *C. parapsilosis* strains. The results are expressed as mean values of three independent experiments (±standard deviation) normalized to the *CpACT1* gene expression level. Figure shows the relative changes in gene expression after 2 h of incubation in the presence of fluconazole (flu), catechin or their combination compared to untreated samples set as 1. * *p* < 0.050 statistically significant; ** *p* < 0.010 highly significant; *** *p* < 0.001 extremely significant.

The presence of catechin induced the expression of transcription factor-encoding gene *CpTAC1* in all three strains tested (Figure 3A–C) and the *CpMRR1* gene only in the CVC and HC clinical isolates (Figure 4A–C). The incubation of HC clinical isolate cells in the presence of catechin and fluconazole leads to a further increase in the expression of both transcription factor genes. Increased *CpTAC1* gene expression in the presence of catechin and fluconazole in both clinical isolates induced the expression of the gene-encoding *Cp*Cdr1p efflux pump only in the HC clinical isolate (Figure 3F). However, the presence of catechin alone or its combination with fluconazole significantly induced the expression of the *CpCDR1B* gene in both *C. parapsilosis* clinical isolates originating from the central venous catheter (CVC) and hemoculture (HC) (Figure 3H,I). The induced expression of the *CpMRR1* gene in the presence of catechin alone or in the presence of catechin with fluconazole correlates with the induced expression of the *CpMDR1* gene in both clinical isolates from the CVC and HC (Figure 4D–F).

**Figure 5:**
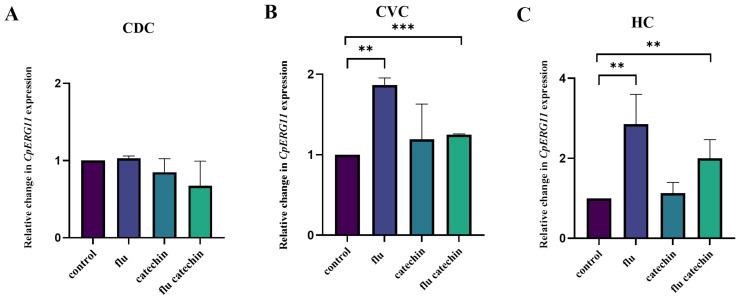
Relative gene expression levels of *CpERG11* gene in the *C. parapsilosis* reference strain CDC317 (**A**) and clinical isolates from CVC (**B**) and HC (**C**). The results are expressed as mean values of three independent experiments (±standard deviation) normalized to the *CpACT1* gene expression level. Figure shows the relative changes in gene expression after 2 h of incubation in the presence of fluconazole (flu), catechin or their combination compared to untreated samples set as 1. ** *p* < 0.01 highly significant; *** *p* < 0.001 extremely significant.

As Figure 5C shows, the presence of fluconazole induced the expression of the *CpERG11* gene in the HC clinical isolate. However, the presence of catechin alone or its simultaneous presence with fluconazole did not lead to a statistically relevant change in *CpERG11* gene expression neither in the CVC clinical isolate nor in the *C. parapsilosis* reference strain CDC317 (Figure 5A,B).

### 2.4. Catechin Reduces the Mortality of Infected Wax Moth Model

**Figure 6:**
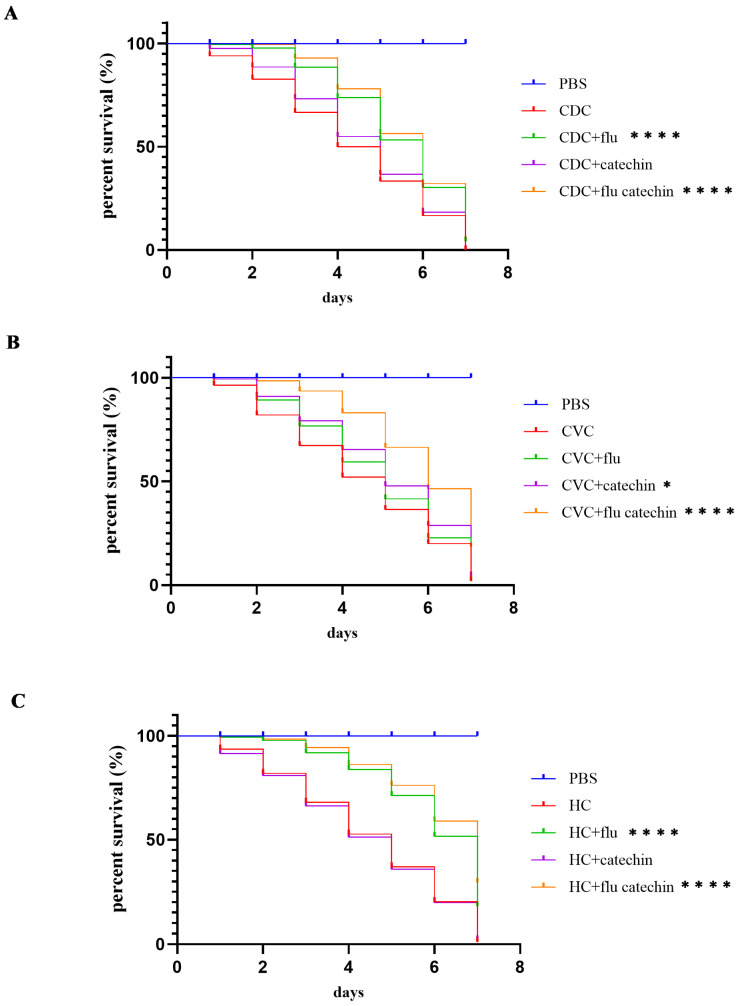
Effect of fluconazole (0.5 mg/mL) and catechin (2 mg/mL) on the survival of *G. mellonella* larvae monitored 7 days after inoculation with *C. parapsilosis* reference strain CDC317 (CDC; (**A**)), CVC clinical isolate (**B**), or HC clinical isolate (**C**). * *p* < 0.050 statistically significant; **** *p* < 0.001 highly extremely significant.

In the final experiment, we compared the survival rates of a *Galleria mellonella* model for fungal infection after infection with the *C. parapsilosis* reference strain CDC317 and the *C. parapsilosis* CVC and HC clinical isolates. We also assessed the viability of *G. mellonella* larvae infected with *C. parapsilosis* strains injected with either fluconazole or catechin alone or with the combination of fluconazole and catechin. The larvae of *G. mellonella* experienced 100% mortality by day 7 post-infection with all *C. parapsilosis* strains. Although the injection of fluconazole alone reduced the mortality of larvae in all strains tested, the simultaneous injection of fluconazole with catechin reduced the mortality of larvae more effectively. On the 7th day post-inoculation, a survival rate 30% was achieved for the *C. parapsilosis* reference strain and CVC clinical isolate. A 50% survival rate of *G. mellonella* larvae was achieved for the *C. parapsilosis* clinical isolate originating from a hemoculture (Figure 6).

## 3. Discussion

*Candida parapsilosis* is a leading cause of invasive candidiasis globally. This yeast species is particularly concerning in hospital environments due to its high transmissibility, which leads to widespread colonization and an increased risk of invasive infections in vulnerable patients. Adding to this concern is a rise in drug resistance. Recent data show that *C. parapsilosis* is developing azole resistance at an alarming rate, with fluconazole resistance being approximately five times more common in this species than in *Candida albicans* [3,24]. Azoles inhibit fungal growth by interfering with the enzyme lanosterol 14α-demethylase (the product of *ERG11*), which converts lanosterol to ergosterol, a key component in the fungal cell membrane. Recent study by Li et al. [25] showed that for azole response in *C. albicans*, interaction between Ncp1p (NADPH-dependent reductase) and Erg11p is necessary. In coupled P450 systems, electrons from NADPH are fully used for substrate hydroxylation, but leakage can produce reactive oxygen species (ROS). Disrupting the Erg11p-Ncp1p interaction in *C. albicans* can serve as a useful approach to enhancing the antifungal properties of azoles. There are two ways that yeast can develop resistance to antifungal drugs. One way is through mutations in transcription factors, for example, in Mrr1p, Tac1p or Upc2p. This causes an increase in the production of the drug’s target protein, which essentially dilutes the antifungal’s effect. Another way is through polyploidy or chromosomal duplication, which also leads to the overexpression of the target protein, making the drug less effective [3,26]. Resistance to azoles in *C. parapsilosis* is mainly caused by amino acid substitutions in the *ERG11* gene, leading to a reduced azole binding affinity [14,27]. Our *C. parapsilosis* clinical isolates possess a Y132F mutation together with an R398I point mutation in the *ERG11* gene [28]. In addition, both *C. parapsilosis* clinical isolates contain two-point mutations, leading to S208G and S304G amino acid substitutions in the *ERG6* gene encoding the sterol C-24 methyltransferase involved in ergosterol biosynthesis. Scarce antifungal arsenal, combined with the rising prevalence of drug-resistant fungal pathogens, represent a significant clinical challenge for the present and future. The use of plants and their bioactive molecules in the treatment of candidiasis has emerged as a promising alternative to traditional drugs, against which resistance has developed in the *Candida* genus [17,22,29]. Catechin exhibits antifungal activity against *C. albicans* by inhibiting its growth and biofilm formation [30]. Catechin also potentiates the effect of fluconazole, inhibiting the growth of fluconazole-resistant *C. albicans*. A combination of miconazole with catechin in *N. glabratus* led to growth inhibition and increased ROS production, resulting in defects in the structure and function of membrane-associated proteins [21]. In this work, we explored the ability of catechin to augment azole susceptibility in two *C. parapsilosis* clinical isolates resistant to fluconazole. We used isolates with sequenced genomes [28]; therefore, in this pioneering study, we employed clinical isolates from the same patient, as well as the reference strain CDC317. On the contrary to *N. glabratus* [21], where catechin alone did not influence their growth, the growth of all *C. parapsilosis* strains tested was reduced in the presence of catechin. Our preliminary experiments showed no synergistic relationship between fluconazole and catechin, however, we show that the administration of catechin together with fluconazole augments the growth inhibition in both *C. parapsilosis* clinical isolates on solid media. This observation corroborates our previous results in *N. glabratus* [22] and those of da Silva et al. [29], who showed that the combination of flavonoids with fluconazole induces apoptotic death in *C. tropicalis*, where the generation and intracellular accumulation of ROS seem to play a crucial role. ROS are a host of unstable molecules that are highly reactive with other molecules in their vicinity, sometimes with detrimental consequences. Synergistic effects have been observed where fluconazole combined with other agents (like osthole or metal ions), which can strongly promote ROS production, even if the drug alone does not [31]. However, despite the accumulating evidence for antifungal drug-induced ROS formation, the implications of this phenomenon are still largely unknown [32]. Catechins appear to be able both to generate and to scavenge free radicals and show their beneficial effects due to a combination of both mechanisms [33,34]. Catechin slightly induced ROS production in both *C. parapsilosis* clinical isolates (CVC and HC) studied and in the CDC317 reference strain. Therefore, induced ROS production by catechin could contribute to the enhanced activity of fluconazole in the presence of catechin. Due to their considerable hydrophobicity, catechins are expected to interact with membranes and penetrate their lipid bilayers. According to our results with *C. parapsilosis* and previous observations for *N. glabratus erg6*Δ mutant [22], we did not observe any statistically significant effect of catechin neither on the plasma membrane fluidity nor on the plasma membrane potential in the *C. parapsilosis* strains studied. In contrast to *C. parapsilosis*, changes in plasma membrane permeability affecting the function of plasma membrane proteins were observed in *N. glabratus* clinical azoles [21].

Although activating mutations in genes encoding transcriptional regulators *Cp*Tac1p and *Cp*Mrr1p are common contributors to fluconazole resistance in *C. parapsilosis*, eliciting their effect through overexpression of efflux pumps, our CVC and HC clinical isolates did not harbor mutations neither in the *CpTAC1* nor in the *CpMRR1* gene. Although the basal expression of the *CpTAC1*, *CpCDR1* and *CpCDR1B* genes in clinical *C. parapsilosis* isolates was significantly lower compared to the reference CDC317 strain (Figure S2), the transcriptional profile of the *C. parapsilosis* CVC and HC clinical isolates in the presence of catechin and also in the presence of catechin together with fluconazole, differed from the transcriptional profile of the reference *C. parapsilosis* CDC317 strain. The genes *CpTAC1* and *CpMRR1*, encoding the transcriptional regulators, were overexpressed in the *C. parapsilosis* CVC and HC clinical isolates, leading to marked overexpression of the *CpCDR1B* gene, encoding *Cp*Cdr1Bp efflux pump and to increased expression of the *CpMDR1* gene, encoding *Cp*Mdr1p transporter belonging to the MFS. As the overexpression of *CpMDR1* in susceptible strains and its disruption in resistant *C. parapsilosis* clinical isolates had little or no effect on fluconazole susceptibility [35], we propose that increased expression of *CpCDR1B* could be responsible for the resistance of our CVC and HC clinical isolates to fluconazole, while increased gene expression may not be reflected in increased function of the gene product. On the contrary, our previous work with *N. glabratus* showed that the presence of catechin lowered the expression of the *CDR1* gene induced by azole to the level observed in control cells [21].

The *CDR1B* locus of *C. parapsilosis* is unique among the CDR genes of the *C. parapsilosis* complex in having two highly similar genes in tandem, which provide a template for the amplification of the locus to occur rapidly. Thus, we cannot exclude that the observed increased expression of *CpCDR1B* is the result of amplification of this locus. Sequencing of the *C. parapsilosis* CDC317 reference strain revealed that the *CDR1B* gene was erroneously assembled by fusing together two highly similar tandem genes, *CDR1B*.1 and *CDR1B*.2 [35]. In the *C. parapsilosis* HC clinical isolate, the simultaneous presence of catechin and fluconazole led also to increased expression of *CpCDR1* gene, encoding *Cp*Cdr1p efflux pump, although Doorley et al. [35] reported that this pump does not have a direct impact on fluconazole susceptibility in *C. parapsilosis*.

Finally, we assessed the potential of catechin in the treatment of *C. parapsilosis* infection in the invertebrate model *G. mellonella*. *G. mellonella* is a useful experimental model, but the main limitation is the difficulty in achieving reproducibility of results compared to the mouse model. This is due to the origin of the larvae, different rearing conditions, storage temperature, nutrition and genetics. To minimize these limitations and improve reproducibility as much as possible, we are maintaining and using our own *G. mellonella* larvae culture. Catechin injection in combination with fluconazole resulted in a reduction in larvae death. Surprisingly, the beneficial effect of catechin with fluconazole was more pronounced in the *C. parapsilosis* HC isolate. This might be related to the fact that catechin increased ROS production with an impact on the survival of the *G. mellonella* larvae infected with *C. parapsilosis* strains. We cannot exclude that the beneficial effect of catechin and fluconazole observed for the HC isolate may be the result of the adaptation of this isolate to stresses, as it is proposed that this strain evolved from the CVC isolate [23]. In this work we used clinical isolates from the same patient; an increased number of clinical isolates and availability of specific *C. parapsilosis* mutant strains would improve the results obtained.

## 4. Materials and Methods

### 4.1. Yeast Strains, Media, Stock Solutions and Oligonucleotides

Two clinical isolates of *C. parapsilosis* [19], HC—isolated from peripheral blood and CVC—isolated from a central venous catheter, were used. *C. parapsilosis* CDC317 was used as the reference strain in all experiments. The MIC 50 values of fluconazole for all strains are listed in Table S1. *C. parapsilosis* cells were plated on YPD medium (1% yeast extract, Biolife, Milan, Italy; 2% peptone, Biolife, Milan, Italy; 2% glucose, Centralchem, Bratislava, Slovakia; 2% agar, Biolife, Milan, Italy). The stock solutions of the tested compounds were as follows: fluconazole (≥98% HPLC powder, Sigma-Aldrich, Burlington, MA, USA) 0.5 mg/mL in distilled water for *Galleria mellonella* infection and 1 mg/mL dissolved in distilled water for other experiments; (+)-catechin-hydrate (≥98% HPLC powder, Sigma-Aldrich, Burlington, MA, USA; further referred as catechin) 10 mg/mL in 20% ethanol, Centralchem, Bratislava, Slovakia; 2′,7′-dichlorofluorescin diacetate (H2DCFDA, Sigma-Aldrich, Burlington, MA, USA) 1 mM in ethanol; diS-C(3) (3,3′-dipropylthiadicarbocyanine, Sigma-Aldrich, Burlington, MA, USA) 0.5 mM in ethanol; DPH (1,6-diphenyl-1,3,5-hexatriene, Serva, Heidelberg, Germany) 0.5 mM in acetone; and TMA-DPH (trimethylammonium diphenylhexatriene, Sigma-Aldrich, Burlington, MA, USA) 0.25 mM in acetone (Centralchem, Bratislava, Slovakia).

The oligonucleotides used in this study are listed in Table S2.

### 4.2. Drug Susceptibility Assays

The susceptibility of the *C. parapsilosis* strains to fluconazole, catechin and their combinations was tested by spot assays. Overnight yeast cultures were grown in YPD medium (1% yeast extract, Biolife, Milan, Italy; 2% peptone, Biolife, Milan Italy; 2% glucose, Centralchem, Bratislava, Slovakia) at 35 °C, 130 rpm, and diluted to a density of 1 × 10^7^ cells/mL. Tenfold serial dilutions were prepared and 5 μL aliquots of each dilution were spotted onto solid YPD plates with various concentrations of the compounds. The concentrations were as follows: fluconazole 25 μg/mL and 50 μg/mL, catechin 2 mg/mL and their combinations. For the control, no further compounds were added to the YPD medium. Colony growth was assessed after 48 h at 35 °C.

### 4.3. Cell Viability Assay

A luminescent assay (BacTiter-Glo^TM^ Microbial Cell Viability Assay, Promega, Fitchburg, WI, USA) was used to measure the viable cells in the culture, where ATP is an indicator of metabolically active cells. Overnight *C. parapsilosis* cultures grown in YPD medium were washed, adjusted to 2.5 × 10^6^ cells/mL and further incubated (35 °C, 130 rpm) with/without fluconazole (8 μg/mL), catechin (2 mg/mL) or their combination for 24 h. All further steps were conducted according to the manufacturer’s protocol. Samples were measured twice with triplicates. The luminescent signal is proportional to the amount of ATP present, which is directly proportional to the number of cells in the culture. The luminescence was measured using a GloMax Discover Microplate Reader (Promega, Fitchburg, WI, USA). For statistical analyses, comparisons with the control were used (unpaired *t*-test).

### 4.4. Isolation of RNA and Quantitative PCR

The relative levels of gene expression were assessed by quantitative PCR (qPCR). Yeast cells were grown in YPD medium to the mid-logarithmic phase. *C. parapsilosis* cells were further cultivated (35 °C, 130 rpm) in YPD broth with/without sub-inhibitory concentrations of fluconazole (5 μg/mL), catechin (2 mg/mL) or their combination for 2 h. The isolation of RNA was performed using the GeneJET RNA Purification Kit (Thermo Scientific, Waltham, MA, USA). Revert AidTM H Minus MMuLV Reverse Transcriptase (Thermo Fisher Scientific, Waltham, MA, USA) was used for first-strand cDNA synthesis, using 1 µg of RNA as the template. qPCR was prepared using the HOT FIREPol^®^ EvaGreen^®^ qPCR Mix Plus (ROX), 5× (Solis Biodyne, Tartu, Estonia, EU). Amplification was performed in the 7900 HT Fast Real-Time PCR System (Applied Biosystems, Foster City, CA, USA). Reporter signals were analyzed using the ABI SDS 2.2.2 software (Applied Biosystems, Foster City, CA, USA). *CpACT1* was used as the reference gene. The gene expression levels were determined according to Livak and Schmittgen [36]. Control samples were normalized to a value of 1. All experiments were performed in three parallel wells and with three independent replicates.

### 4.5. Detection of Intracellular ROS Levels

The production of ROS was measured using H2DCFDA (Sigma-Aldrich, Burlington, MA, USA). Cells were grown in YPD. A total of 1 × 10^7^ cells/mL were pretreated for 2 h at 35 °C with catechin (2 mg/mL), fluconazole (5 μg/mL, sub-inhibitory concentration) or a combination of both in YPD medium. The cells were washed in PBS (Sigma-Aldrich, Burlington, MA, USA, pH 7) and 1 × 10^6^ cells were incubated with 25 μM H2DCFDA in a black 96-well plate, as previously described in Tóth Hervay et al. [21]. As a negative control, cells in PBS with no H2DCFDA added were used. The DCF (2′,7′-dichlorofluorescein) fluorescence signal was measured using a GloMax Discover Microplate Reader (Promega Corp., Madison, WI, USA) after 90 min at excitation and emission wavelengths of 475 and 500–550 nm. For statistical analyses, comparisons with the control (cells incubated without xenobiotics) were used (unpaired *t*-test).

### 4.6. Fluorescence Anisotropy and Plasma Membrane Potential Measurements

The *C. parapsilosis* cells grown overnight in YPD medium at 35 °C were further cultivated (35 °C, 130 rpm) in YPD medium with or without fluconazole (5 μg/mL), catechin (2 mg/mL) or their combination for 2 h. The cells were washed twice in Tris–Cl buffer (10 mmol/L Tris base Sigma-Aldrich, Burlington, MA, USA; pH 7.0 adjusted with HCl, Centralchem, Bratislava, Slovakia) and labeled with DPH (2 μM, 1,6-diphenyl-1,3,5-hexatriene, Sigma-Aldrich, Burlington, MA, USA) or TMA-DPH (2 µΜ, 1-(4′-trimethylammoniumphenyl)-6-phenyl-1,3,5-hexatriene, Sigma-Aldrich, Burlington, MA, USA) for plasma membrane fluidity determination or stained with the potentiometric fluorescent probe diS-C3(3) (2 μM, 3,3′-Dipropylthiadicarbocyanine, Sigma-Aldrich, Burlington, MA, USA) to measure plasma membrane potential, as previously described in Toth Hervay et al. [37]. Plasma membrane fluidity was determined using the Luminescence Spectrometer Perkin Elmer LS 55 with an L-format measurement (Perkin Elmer, Waltham, MA, USA) at 22 °C. The excitation wavelength was 360 nm and the emission wavelength was 430 nm. For statistical analysis, an unpaired *t*-test was used.

### 4.7. Galleria mellonella Infection

Antifungal effectivity of catechin in vivo was evaluated by using *Galleria mellonella* model. Overnight, *C. parapsilosis* cultures were washed twice with PBS (1×, pH 7.2) and adjusted to 4 × 10^8^ cells/mL. For the survival test, larvae with a weight of 300 mg were selected. Groups (*n* = 15) of 20 randomly selected larvae were infected with 10 μL of yeast cultures via the last left proleg. Treated groups were also injected with fluconazole (16.6 mg/kg), catechin (66.6 mg/kg) or their combination into the last right proleg. Larvae injected with PBS were used as negative control groups to monitor the trauma of injection (*n* = 20). Larvae were incubated at 35 °C for 7 days. Survival was monitored every 24 h by visual inspection.

### 4.8. Statistical Analysis

For statistical analysis, an unpaired *t*-test was performed using GraphPad Prism 10 software (GraphPad, San Diego, CA, USA) comparing the two separate groups (treated vs. control group). For the cell viability assay and detection of ROS levels, at least two biological replicates, each measured in triplicate, were analyzed. For fluorescence anisotropy and plasma membrane potential measurements, at least ten biological replicates were analyzed. The statistical significance of qPCR data was assessed with the Kruskal–Wallis test using GraphPad Prism software (GraphPad, San Diego, CA, USA). Survival curves of infected *Galleria mellonella* larvae were analyzed using GraphPad Prism 10 (GraphPad Software, San Diego, CA, USA). Statistical significance was determined via the log-rank (Mantel–Cox) test, with each experimental group compared against the control group (PBS). Differences were considered significant at various *p*-values: *p* > 0.050 was considered non-significant, *p* < 0.050 (*) was significant, *p* < 0.010 (**) was very significant, *p* < 0.001 (***) was highly significant and *p* < 0.0001 (****) was extremely significant.

## 5. Conclusions

This study provides the first comprehensive evaluation of catechin’s antifungal potential against *C. parapsilosis.* Notably, our findings reveal a distinct mechanism of action that differs significantly from the previously reported effects in other fungal species, highlighting the unique physiological response of *C. parapsilosis* to catechin treatment. Our results demonstrate that catechin itself affects the growth of cells in the monitored strains of *C. parapsilosis*. The production of ROS was not as pronounced as in strains of *N. glabratus*, and no change in membrane fluidity or membrane potential was observed after exposure to catechin. Significant changes in the expression of genes associated with fluconazole resistance in *C. parapsilosis* indicate a different response to catechin at the level of gene expression, as was already observed in other yeast species of the genus *Candida*. Further studies are needed to clarify the differences in the mechanisms of action of catechin on *C. parapsilosis.* We propose that future studies using mutants in transcription factors and transport proteins, which are essential for clarifying the function of efflux pumps in *C. parapsilosis*, are necessary. Experiments analyzing the substrate specificity of the pumps would also be useful.

## Data Availability

The original contributions presented in this study are included in the article/Supplementary Materials. Further inquiries can be directed to the corresponding author.

## Supplementary Materials

The following supporting information can be downloaded at: https://www.mdpi.com/article/10.3390/ijms27020620/s1.

## Abbreviations

The following abbreviations are used in this manuscript:

ABC	ATP-Binding Cassette
CVC	Central Venous Catheter
DCF	2′,7′-dichloroluorescein
H2DCFDA	2′,7′-dichlorodihydrofluorescein diacetate
HC	Hemoculture
MDR	Multi-Drug Resistance
MFS	Major Facilitator Superfamily
PBS	Phosphate-Buffered Saline
