# Supplementary material for: Catechin Augments the Antifungal Efficacy of Fluconazole Against Candida parapsilosis
Source: Int J Mol Sci. 2026 Jan 7;27(2):620. doi: 10.3390/ijms27020620 (PMC12840672; doi:10.3390/ijms27020620)
Supplement: Supplementary file 1 [file ijms-27-00620-s001.zip › Table S3.pdf]

Table S3. Influence of fluconazole and catechin on the values of fluorescence anisotropy in the *C. parapsilosis* CDC317 cells. Values represent the mean of 10 independent biological replicates

CDC317	DPH	TMA-DPH
control	0.292±0.012	0.354±0.007
fluconazole	0.299±0.011	0.362±0.003
catechin	0.295±0.009	0.3605±0.002
fluconazole + catechin	0.300±0.007	0.358±0.006
