# Supplementary material for: Catechin Augments the Antifungal Efficacy of Fluconazole Against Candida parapsilosis
Source: Int J Mol Sci. 2026 Jan 7;27(2):620. doi: 10.3390/ijms27020620 (PMC12840672; doi:10.3390/ijms27020620)
Supplement: Supplementary file 1 [file ijms-27-00620-s001.zip › Table S2.pdf]

Table S2. List of oligonucleotides used in this study

**Primers for qRT-PCR analysis**	**Nucleotide sequence (5′ → 3′)**
CpACT1- F	TTGCTCCAGAAGAACACCCA
CpACT1- R	TGACACCATCACCTGAATCCA
CpCDR1- F	ACAGTCACAAATGCACTTTGGA
CpCDR1- R	ATGTAGAACAACCGGCACCT
CpCDR1B- F	AAGCAGCAAACTTCTTCCTC
CpCDR1B- R	TGAAGTGGTATCCTCATTTGTC
CpMDR1- F	TCCCCATTGCTATTGTTGGT
CpMDR1- R	TGCGCCCATATAATTGAACA
CpTAC1- F	ATCAGATGCAGGTCATAGAGGT
CpTAC1- R	GCTCCATCCAATCCAACGA
CpMRR1- F	CAACATCAACATCAACGCC
CpMRR1- R	TGCGCCCATATAATTGAACA
CpERG11- F	TGTTGCATTTGGCTGAGAAG
CpERG11- R	TCTGAGGGTTTCCTTGATGG
