# Supplementary material for: Catechin Augments the Antifungal Efficacy of Fluconazole Against Candida parapsilosis
Source: Int J Mol Sci. 2026 Jan 7;27(2):620. doi: 10.3390/ijms27020620 (PMC12840672; doi:10.3390/ijms27020620)
Supplement: Supplementary file 1 [file ijms-27-00620-s001.zip › Table S1.pdf]

Table S1. Susceptibility of *Candida parapsilosis* strains to fluconazole

	MIC_{50} (µg/mL)	
	Fluconazole	
	YPD	RPMI-1640
**CDC317**	**16**	**8**
**CVC**	**32**	**16**
**HC**	**32**	**16**
