# Supplementary figures and images for: Catechin Augments the Antifungal Efficacy of Fluconazole Against Candida parapsilosis
Source: Int J Mol Sci. 2026 Jan 7;27(2):620. doi: 10.3390/ijms27020620 (PMC12840672; doi:10.3390/ijms27020620)

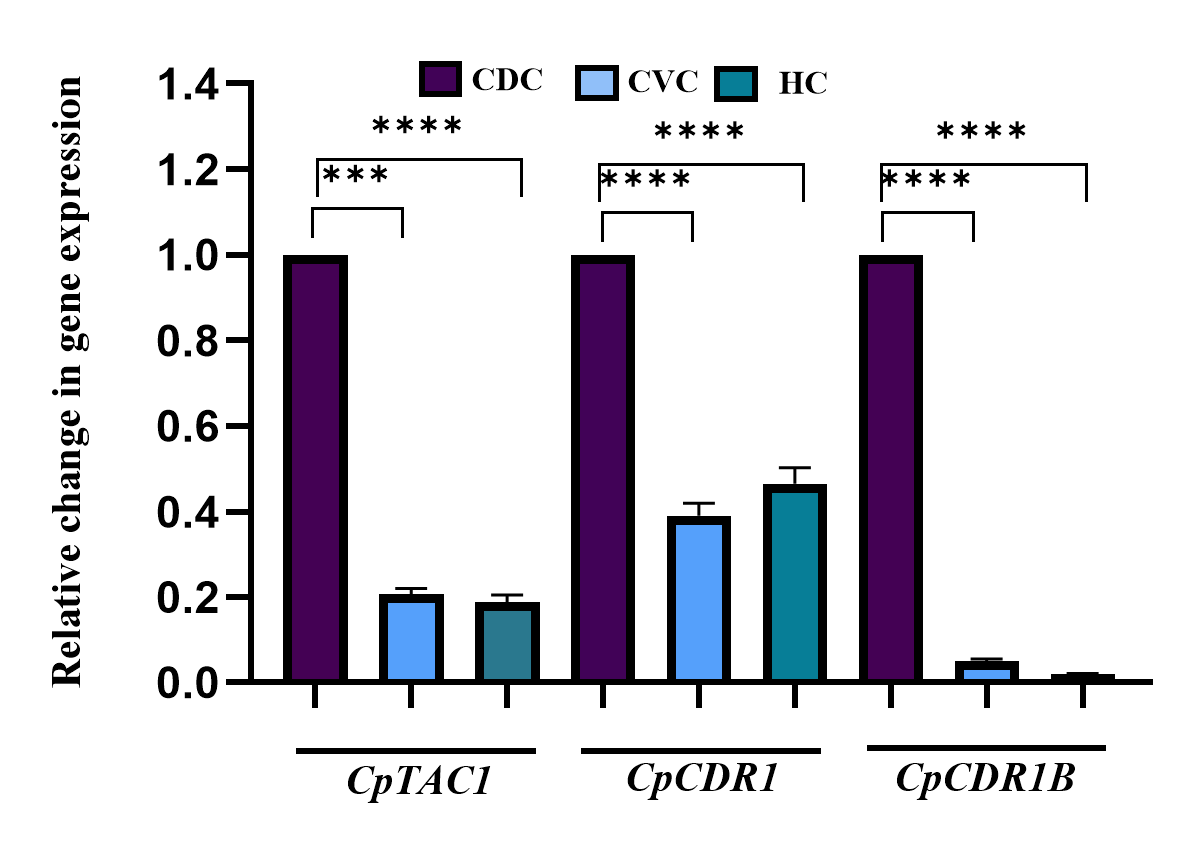

Supplement: Supplementary file 1 [file ijms-27-00620-s001.zip › Figure S2.tif]

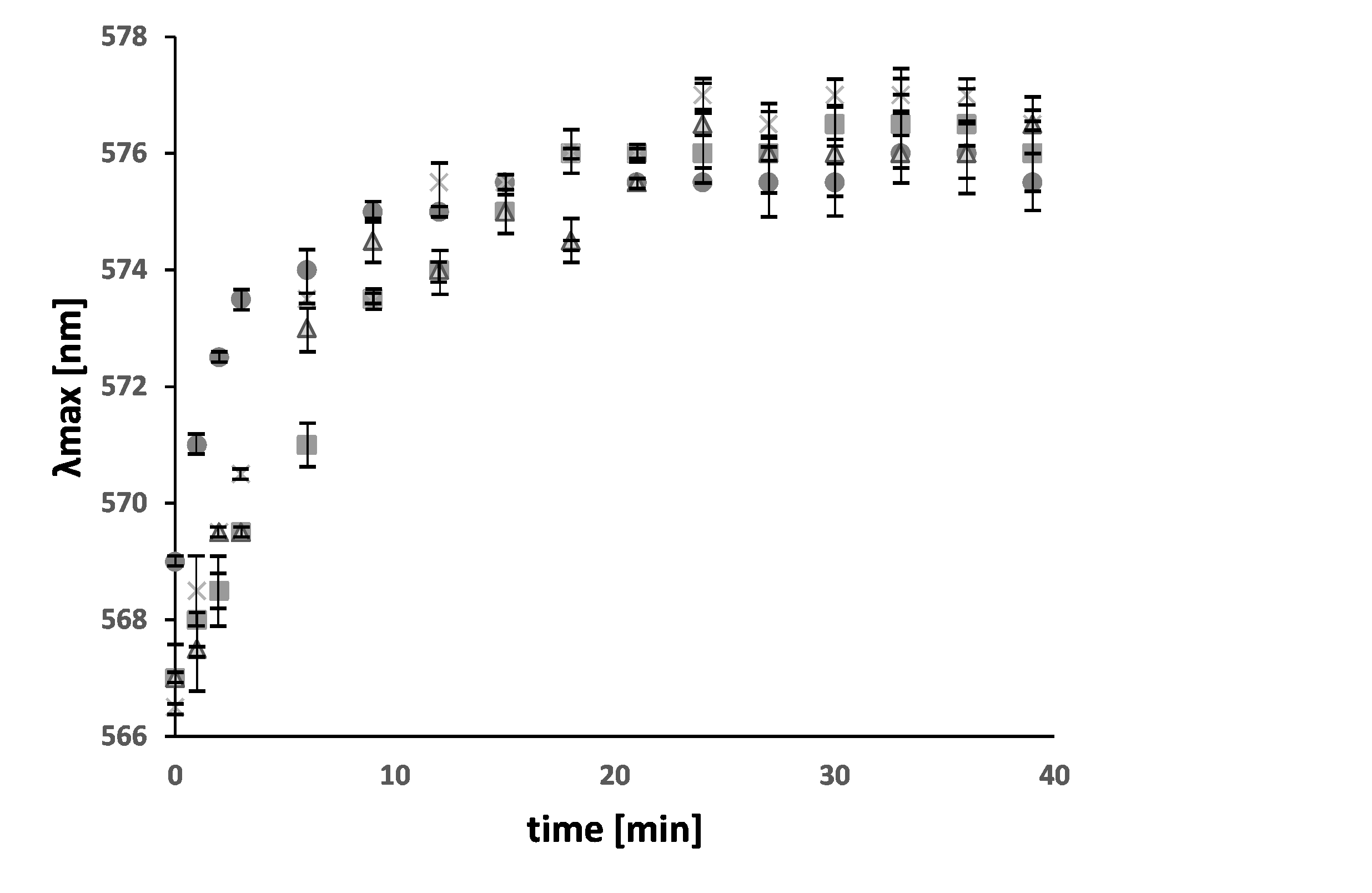

Supplement: Supplementary file 1 [file ijms-27-00620-s001.zip › Figure S1. IJMS.tif]
